# MMP14 in Sarcoma: A Regulator of Tumor Microenvironment Communication in Connective Tissues

**DOI:** 10.3390/cells8090991

**Published:** 2019-08-28

**Authors:** Jordi Gonzalez-Molina, Silvia Gramolelli, Zehuan Liao, Joseph W. Carlson, Päivi M. Ojala, Kaisa Lehti

**Affiliations:** 1 Department of Microbiology, Tumor and Cell Biology (MTC), Karolinska Institutet, 17177 Stockholm, Sweden; 2 Department of Oncology-Pathology, Karolinska Institutet, 17176 Stockholm, Sweden; 3 Translational Cancer Medicine Research Program, Faculty of Medicine, University of Helsinki, 00014 Helsinki, Finland; 4 School of Biological Sciences, Nanyang Technological University Singapore, 60 Nanyang Drive, Singapore 637551, Singapore; 5 Section of Virology, Division of Infectious Diseases, Department of Medicine, Imperial College London, London W2 1NY, UK; 6 Individualized Drug Therapy Research Program, Faculty of Medicine, University of Helsinki, 00014 Helsinki, Finland

**Keywords:** MMP14, sarcoma, tumor microenvironment, mesenchymal phenotype, metastasis

## Abstract

Sarcomas are deadly malignant tumors of mesenchymal origin occurring at all ages. The expression and function of the membrane-type matrix metalloproteinase MMP14 is closely related to the mesenchymal cell phenotype, and it is highly expressed in most sarcomas. MMP14 regulates the activity of multiple extracellular and plasma membrane proteins, influencing cell–cell and cell–extracellular matrix (ECM) communication. This regulation mediates processes such as ECM degradation and remodeling, cell invasion, and cancer metastasis. Thus, a comprehensive understanding of the biology of MMP14 in sarcomas will shed light on the mechanisms controlling the key processes in these diseases. Here, we provide an overview of the function and regulation of MMP14 and we discuss their relationship with clinical and pre-clinical MMP14 data in both adult and childhood sarcomas.

## 1. Introduction

Sarcomas are a heterogeneous and deadly group of mesenchymal malignancies that are relatively rare, accounting for <1% of all adult malignancies, but they are the fourth most common childhood cancer type [1]. Sarcomas can occur at all sites of the body and are generally classified as sarcomas of the soft tissue or bone (i.e., osteosarcoma) depending on the connective tissue they originate from. However, the identification of molecular and histological traits of the different subtypes rather than the tissue of origin are stronger parameters for the classification of sarcomas [2]. Thus, understanding the biological mechanisms behind these histological characteristics will be essential for improving their clinical management.

The crosstalk between the surrounding tumor microenvironment (TME) and sarcoma cells plays a central role in tumor initiation and progression, affecting patient prognosis [3]. The extracellular matrix (ECM), including fibrillar collagens as the major connective tissue components, is often aberrantly regulated in sarcomas, and the increased matrix deposition, crosslinking, and degradation are all characteristics that make sarcomas aggressive tumors [4,5,6]. Neighboring cells such as endothelial cells, immune cells and cancer associated fibroblasts represent the other main component of the TME. These cells are also involved in and affected by ECM remodeling, altogether contributing to sarcoma metastasis and response to therapies [7,8]. Understanding the differential characteristics of the communication between cancer cells and the TME will open new avenues for sarcoma prognosis and treatment.

Matrix metalloproteinases (MMP) are a group of proteolytic enzymes that mediate several of the changes in the TME occurring during tumor development and progression [9]. In humans, 6 different membrane-type matrix metalloproteinases (MT-MMPs) have been described. These include four type I MT-MMPs anchored to the plasma membrane through a transmembrane domain, namely MT1-MMP (MMP14), MT2-MMP (MMP15), MT3-MMP (MMP16), and MT5-MMP (MMP24), as well as the glycosylphosphatidylinositol-anchored MT4-MMP (MMP17) and MT6-MMP (MMP25) [10]. MMP14 was first described by Sato et al. as a transmembrane protein which activates pro-MMP2 to induce tumor cell invasion [11]. Most MMPs are secreted as inactive pro-proteinases that are activated by proteolytic cleavage. Active MMP14 binds to the metallopeptidase inhibitor, tissue inhibitor of metalloproteinases 2 (TIMP2), to form a receptor for proMMP2 activation [12,13]. MMP14 knockout mice exhibit defects in skeletal development and angiogenesis, fibrosis of soft tissues, and premature death. This phenotype has been attributed largely to the importance of MMP14 in collagen turnover and bone remodeling [14,15]. While mice deficient in MMP2 exhibit relatively mild skeletal defects which result in reduced bone mineralization and joint erosion, mutations in human MMP2 and MMP14 cause the severe connective tissue syndromes known as MONA (Multicentric Osteolysis, subcutaneous Nodulosis, and Athropathia) and Winchester Syndrome, respectively [16,17,18,19].

**Figure 1:**
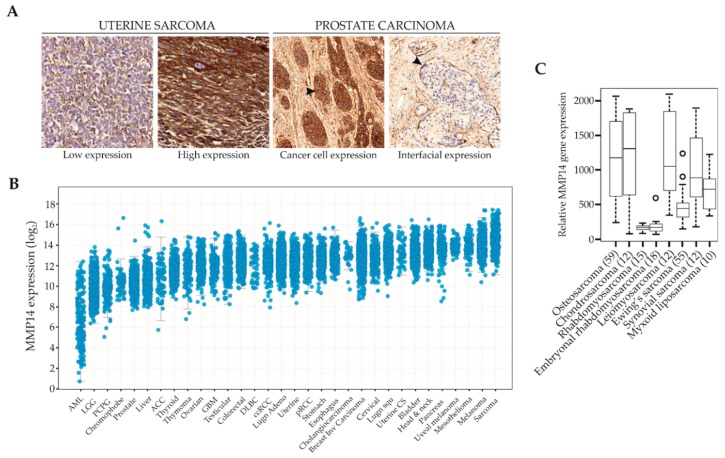
MMP14 expression in cancer. (**A**): Examples of MMP14 protein expression (in brown) in sarcoma (uterine sarcoma) and carcinoma (prostate carcinoma) tumors. Whereas in epithelial cancers, either the tumor cells or adjacent cancer associated fibroblasts can express MMP14, the mesenchymal sarcoma cells themselves express the protease. Arrowheads indicate high MMP14-expressing regions. (**B**): *MMP14* gene expression in various cancer types based on The Cancer Genome Atlas program (TCGA) (www.cbioportal.org/) [28,29]. (**C**): *MMP14* gene expression in various types of sarcoma (http://ist.medisapiens.com/) [30].

MMP14 is up-regulated in several types of cancer, promoting angiogenesis, inflammation, cancer cell invasion, and metastasis (Figure 1A) [20,21,22,23]. In genetically-modified mouse models, MMP14 overexpression induces mammary gland adenocarcinoma formation and pancreatic cancer development [24]. Other mouse models of epithelial cancers have also identified MMP14 expression, particularly in tumor-associated cells of the TME, to be involved in cancer progression. An MMP14-deficient breast cancer mouse model showed reduced metastasis; an effect attributed to the reduced collagen I degradation by stromal fibroblasts [25]. Similarly, normal mammary gland branching depends on the expression of MMP14 by stromal but not mammary epithelial cells [26]. In addition, tumor-associated macrophages express MMP14 and are involved in matrix remodeling, as shown in a colorectal cancer orthotopic mouse model [27]. Surprisingly, while MMP14 in the context of cancer has mostly been investigated in epithelial tumors, its role in sarcoma remains relatively unexplored. Yet, the MMP14 gene expression across a variety of cancer types is highest in sarcomas, with the childhood rhabdomyosarcomas and Ewing sarcoma representing intriguing exceptions (Figure 1B,C, www.cbioportal.org/), suggesting that it may be a particularly important player in sarcoma biology [28,29].

## 2. Activity of MMP14

In addition to pro-MMP2, other proteases have been identified as MMP14 substrates such as the zymogens pro-MMP8 and pro-MMP13 [31,32]. Moreover, MMP14 not only induces the proteolysis of collagen I but is also involved in the degradation of various other ECM components such as collagens II–IV, gelatins, fibronectin, tenascin, laminins, fibrin, vitronectin, nidogen, and aggrecan [33,34]. The cleavage of ECM components also leads to the release and modification of biologically active molecules such as growth factors and cytokines including the transforming growth factor (TGF)-beta [35]. Furthermore, MMP14 processes latent TGF-beta-binding protein 1 and pro-TGF-beta as well as soluble chemokines such as the stromal cell-derived factor (SDF)-1 and the monocyte chemoattractant protein (MCP)-3, having a direct effect on the immune system [36,37,38].

Processing and shedding of membrane-bound proteins is another major function of MMP14. Several adhesion molecules are among these proteins, including the ECM-binding integrins αv and α5, by which MMP14 affects cell motility [39,40]. The adhesion of integrins to fibronectin is modulated by tissue transglutaminase, which is an MMP14 substrate [41]. In addition, shedding of the ectodomain of the hyaluronic acid receptor CD44 by MMP14 induces cell migration [42,43]. Other membrane-anchored proteins affected by MMP14 include the low density lipoprotein receptor-related protein (LRP), Syndecan-1, ephrin type-A receptor 2 (EphA2), the transmembrane mucin MUC-1, and the extracellular matrix metalloproteinase inducer (EMMPRIN), among others [44,45,46,47,48,49]. Moreover, MMP14 soluble form results from an autocatalytic process [10].

MMP14 also has non-proteolytic functions such as the TIMP2-dependent activation of the Ras-Raf-ERK signaling cascade, which is mediated by the cytoplasmic tail of MMP14 through a process that involves the physical association between MMP14 and β1 integrin [50,51]. Moreover, MMP14 is required for lamellipodia formation and motility of myeloid progenitors, a process dependent on the MMP14 cytoplasmic domain, which activates the Rho GTPase Rac1 through its association with the adaptor protein p130Cas [52]. In addition, both β1-integrin activation and Notch3 expression depend on the MMP14 relocalization to the plasma membrane in melanoma cells upon contact with lymphatic endothelial cells, which triggers an enhanced 3D invasive sprouting of the tumor cells [53].

## 3. MMP14 and the Mesenchymal Phenotype

Mesenchymal cells are characterized by the lack of apical-basal polarity, typically presenting a spindle shape, capacity for high motility, front-rear polarity, and high ECM-remodeling capabilities. In line with their ECM-remodeling characteristics, these cells typically express high levels of MMP14. According to the Medisapiens database (http://ist.medisapiens.com/), mesenchymal stem cells are, indeed, among the non-pathological cell types with highest MMP14 gene expression [30]. Moreover, during development, cells of mesenchymal origin specifically express MMP14 [54].

The processes known as epithelial- and endothelial-to-mesenchymal transition, where epithelial or endothelial cells acquire mesenchymal features, occur both in physiological contexts like development and wound healing as well as in pathological processes such as cancer. The induction of epithelial-to-mesenchymal transition (EMT), regulated by the major EMT-associated transcription factors SNAI, TWIST, and ZEB, is accompanied by the upregulation of MMP14 expression, suggesting a close relationship between the mesenchymal phenotype and MMP14 [10]. Furthermore, enhanced expression of MMP14 has been reported to induce the acquisition of a mesenchymal phenotype in cancer and during development, in part due to its function in cleaving collagen IV and laminins of the epithelial basement membrane as well as the cell-cell junction protein E-cadherin [23,55,56,57,58,59,60].

Given the heterogeneity of sarcoma tissues, the phenotype of sarcoma cells can also vary, with cells presenting mesenchymal, epithelial, and mesenchymal-epithelial mixed characteristics. Interestingly, the process termed mesenchymal-to-epithelial transition (MET) has been reported in several soft tissue sarcomas [61], but the regulation of MMP14 during this process has not yet been described. However, during somatic reprogramming of mouse embryonic fibroblasts to pluripotency, a MET-like process occurs together with the downregulation of MMP14 protein expression suggesting a link between this process and MMP14 [62]. In synovial sarcoma and leiomyosarcoma, the downregulation of SNAI transcription factors induces an epithelial phenotype [63,64]. Moreover, transcriptomic data from the Cancer Genome Atlas program (TCGA) shows a significant correlation between the expression of *MMP14* and the transcription factors *TWIST* (*TWIST1*: r = 0.4, *p* = 8.31 × 10^−11^; *TWIST2*: r = 0.21, *p* = 9.72 × 10^−4^) and *SNAI* (*SNAI1*: Pearson’s r = 0.23, *p* = 3.08 × 10^−4^; *SNAI2*: r = 0.21, *p* = 9.96 × 10^−4^) in sarcomas, suggesting that MMP14 expression is coupled with the transcriptional program governing the sarcoma phenotype. Experimentally, MMP14 overexpression in the synovial sarcoma cell line SW982 has been shown to induce spindle shape morphology and an EMT-like phenotype, in conjunction with enhanced cell invasiveness [65]. Together, these reports establish the tight association between MMP14 and the mesenchymal phenotype, suggesting that MMP14 may also contribute to determining the phenotypical characteristics of sarcomas. However, the regulatory interrelationships between MMP14 and the EMT or MET processes remain poorly understood.

## 4. MMP14 in Sarcoma

### 4.1. Expression of MMPs in Soft Tissue Sarcomas

The use of *MMP* gene and/or protein as diagnostic markers has resulted in seemingly contradictory results. Copy number alterations in sarcomas are generally rare. Only 1.3% of general soft tissue sarcomas present gains of the *MMP14* gene. However, when considering only angiosarcoma, 12.5% of patients present increased copy numbers of this gene (www.cbioportal.org/) [28,29]. In other sarcomas, the increased *MMP14* expression is a far more common phenomenon than copy number alterations. By comparing the *MMP14* expression in tumor and matching normal tissues in a pan-sarcoma cohort, a general increase in *MMP14* is observed in sarcomas (www.gepia.cancer-pku.cn/). Moreover, an even more pronounced *MMP14* expression is induced in both leiomyosarcoma and liposarcoma (www.oncopression.com/).

Gene expression of *MMPs* in general associates poorly with disease prognosis. The gene expression for non-membrane type, secreted MMPs, shows no association with disease-free survival in undifferentiated pleomorphic sarcoma, liposarcoma, and synovial sarcoma, except for MMP8 and MMP13 [66]. Moreover, no significant differences have been observed in the expression of MMP14 between non-metastatic and metastatic undifferentiated pleomorphic sarcoma (UPS) groups [67]. On the other hand, we reported that an aggressive subgroup of undifferentiated uterine sarcoma cases, presenting the poorest survival rates of all subgroups, is characterized by high *MMP14* gene and protein expression [68].

Studies investigating the protein expression and activity of MMP14 and other MMPs, including MMP2, have found stronger associations with disease prognosis, grade, and histopathological features. In UPS, MMP2 and MMP9 protein expression and activity are higher in malignant tissues than in the normal counterparts, and both the pro- and active forms of MMP2 are increased in metastatic compared to non-metastatic patients [67]. Also in UPS, cells with pleomorphic characteristics (spindle cells admixed with other mesenchymal elements) are associated with active MMP1 and MMP9 expression compared to tissues consisting purely of spindle cells, whereas MMP2 levels appear to be unaffected [69]. In synovial sarcoma, MMP14 protein expression is higher in TNM stages III and IV than in stages I and II, and the expression of MMP14 correlates with that of EMT-related proteins such as increased N-cadherin and decreased E-cadherin. Moreover, high MMP14 expression is mainly observed in spindle cell monophasic fibrous synovial sarcomas [65]. In synovial sarcoma and liposarcoma, enhanced MMP2 protein also correlates with poor disease-free survival and in liposarcoma it correlates with both tumor grade and metastasis [70]. In a cohort of various sarcoma types as well as benign soft tissue neoplasms, MMP14 protein expression has been found to be higher in sarcomas than in the benign tissues [71]. The levels of MMP2 in blood vessels are also lower in benign neoplasms, and the percentage of activated MMP2 correlates with tumor size [71]. In this study, MMP14 expression, however, does not correlate with active MMP2, although MMP14 activity or the presence of other MMP2-modulatory proteins such as TIMP2 are not considered. The authors, thus, argue that the high tissue expression of MMP14 may contribute to the activation of vascular MMP2, promoting angiogenesis [71].

These studies highlight the differences between MMP14 gene and protein expression, as well as the activity of MMP14 and MMP2, indicating that MMP14 protein level and activity are better prognostic factors than the corresponding gene expression. This poor correlation can be explained by the multiple post-transcriptional mechanisms that regulate the activity of MMPs (discussed below). Moreover, understanding the cell phenotype- and tissue-specific functions of MMP14 in sarcoma will be central to evaluate its potential as a prognostic marker or a therapeutic target.

### 4.2. MMP14 in Childhood Sarcomas

The most common soft tissue sarcomas occurring primarily in children or young adults are rhabdomyosarcoma and Ewing sarcoma. Rhabdomyosarcoma is generally divided into three main histological subtypes, the most common being embryonal, followed by alveolar and anaplastic. Ewing sarcoma is a type of small round cell sarcoma that can originate both in bone and soft tissues and is characterized by presenting FET-ETS gene fusions [72]. Few clinical studies have investigated the role of MMP14 in rhabdomyosarcoma and Ewing sarcoma, which both express relatively low levels of MMP14 as compared to other types of sarcoma (Figure 1C). However, some reports suggest that MMP14 could also be important in specific subtypes of these malignancies [73,74,75]. For instance, the more aggressive alveolar rhabdomyosarcoma presents higher protein levels of both MMP14 and MMP2 than the embryonal rhabdomyosarcoma type, where both these proteases are often undetectable [73]. In Ewing sarcoma cell lines, a peculiar repertoire of MMP expression has been reported, with very low *MMP1* and *MMP3* mRNAs due to the presence of fusion proteins of promoter trans-activators of MMPs, resulting in a drastic reduction of their transcription. However, all cell lines used in this study expressed both MMP2 and MMP14, suggesting that MMP14 influences Ewing sarcoma cell behavior [74]. Another piece of evidence for MMP14 activity in Ewing sarcoma is that the same cells expressing MMP14 protein also present soluble Endoglin, a membrane coreceptor of the TGFβ family, which is cleaved by MMP14 and associates with poor prognosis in Ewing sarcoma [75].

### 4.3. MMP14 in Kaposi Sarcoma

Kaposi sarcoma (KS) is an angiogenic tumor of endothelial origin arising in severely immunocompromised individuals infected with Kaposi sarcoma herpesvirus (KSHV), the causative agent of this cancer. Interestingly, KSHV infection of lymphatic endothelial cells grown in 3-dimensional (3D) matrix induces endothelial-to-mesenchymal transition (EndMT), thus skewing the lymphatic endothelial cell identity towards that of a mesenchymal cell type with enhanced invasive properties. The upregulation of mesenchymal markers has been observed also within KS tissues in the virus-positive tumor cells [57]. The increased cell invasion of KSHV-infected endothelial cells has been linked to the systematically increased expression of several MMPs e.g., MMP1, MMP7, MMP13, and MMP14 [76]. MMP14, whose prominent expression is also seen in the virus-infected cells within KS tumors, was specifically identified as a key player for the virus-induced sprouting of KSHV-infected lymphatic endothelial cells in a 3D fibrin matrix and for the endothelial-to-mesenchymal reprogramming of KSHV-infected lymphatic endothelial cells [57].

### 4.4. MMP14 in Osteosarcoma

Osteosarcomas are the most common bone tumors, mainly affecting children and adolescents. Osteosarcomas are composed of malignant cells producing immature bone or osteoid tissue containing primarily collagen I, which suggests that collagen remodeling might take part in the development of these tumors [77]. Osteosarcoma cells express high levels of MMP14 mRNA, and the corresponding protein correlates with poor prognosis in patients [78]. Moreover, high levels of EMMPRIN, an MMP14 substrate that stimulates the expression of MMPs [79], as well as the co-expression of EMMPRIN and MMP14, predict poor prognosis [80]. The comparison of the gene expression between osteosarcoma samples and matching normal bone tissue also reveals *MMP14* as one of the most significantly upregulated genes [81]. Clinically, an elevated MMP2/MMP9 activity ratio, but not mRNA or protein expression, associates with poor response to chemotherapy in osteosarcoma [82]. In addition, the destruction of bone tissue caused by osteosarcoma invasion is regulated by MMP14 activity together with the endocytic collagen receptor uPARAP/Endo180 in osteosarcoma cells, a process that, contrary to bone metastases of epithelial cancers, does not require osteoclast activity [83].

## 5. Regulation of MMP14 Expression and Activity

MMPs are enzymes that efficiently degrade the ECM. Therefore, high MMP levels may lead to detrimental effects on tissue architecture and homeostasis. To avoid this, multiple layers of regulation for the expression of each MMP exist, and their regulation is influenced by both intra- and extra-cellular cues.

### 5.1. MMP14 Transcriptional Regulation

**Figure 2:**
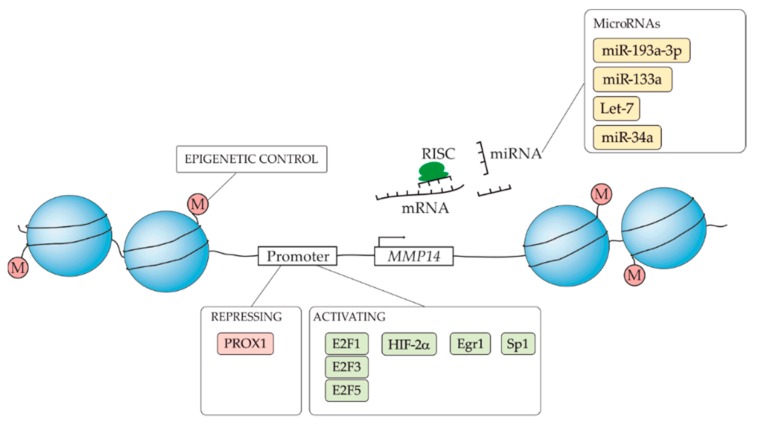
MMP14 is regulated at the transcriptional level by multiple activators (highlighted in green) and a repressor (in red). DNA methylation (M) in the promoter region also regulates the transcription of the *MMP14* gene. At the first post-transcriptional level, various microRNAs (in yellow), including several microRNAs with reported activities in various types of sarcoma, have been shown to target and thus reduce the translation of MMP14 mRNA.

MMP14 expression is tightly regulated at the transcriptional and epigenetic levels (summarized in Figure 2). The *MMP14* promoter exhibits distinctive features, which render it unique compared to most other MMPs. For instance, it lacks a TATA-box and harbors a functional, although non-conventional, Sp1 transcription factor (TF) binding site [84]. An extensive characterization of the *MMP14* promoter points to at least five different transcription start sites (TSS) and the presence of a repressive regulatory element located between −1200 and −385 nt upstream of the main TSS [84]. Recently we have identified PROX1, the master regulator of lymphatic endothelial cell development and a TF involved in differentiation of organs such as liver, pancreas, retina, and brain [85], as the first direct repressor of MMP14 transcription [86]. Notably, PROX1 binds to the *MMP14* promoter at two specific, adjacent regions located within the previously identified repressive regulatory region [84]. The PROX1-MMP14 axis represents a regulatory mechanism of cancer cell invasion and endothelial cell specification [86]. Amongst sarcomas, PROX1 is highly expressed in rhabdomyosarcoma, which could explain the generally low expression of MMP14 in this sarcoma type. Moreover, in KS tumors PROX1 positive cells do not express MMP14 and, vice versa, MMP14-expressing cells lack PROX1 expression [86]. Interestingly, KSHV-infection induces PROX1 transcriptional downregulation in lymphatic endothelial cells [87], which can, thus, likely contribute to the prominent MMP14 expression in KS tumors. In support of this is also our unpublished RNA-seq data, where depletion of PROX1 in KSHV-infected lymphatic endothelial cells drives a significant increase in MMP14 transcript levels.

In the context of epithelial tumor invasion, renal cell carcinoma patients with increased levels of HIF2α, due to the genetic loss of the ubiquitin-ligase VHL, also display increased MMP14 levels and higher metastatic rates [88]. Mechanistically, HIF2α, in concert with Sp1, can bind to the MMP14 promoter thereby enhancing its expression and the invasiveness of cancer cells. Upon tetraspanin CD81 stimulation, MMP14 expression as well as MMP14-dependent melanoma invasion and metastasis are increased through Akt-dependent Sp1 activation, which also support the function of Sp1 in regulating the transcription of MMP14 [89].

Notably, all 23 human *MMP* gene promoters harbor an E2F binding site but, to date, only MMP9, MMP14, and MMP15 have been shown to respond to this transcription factor [90], with E2F1, E2F3, and E2F5 being involved in the transcriptional induction of the *MMP14* gene. E2F levels are tightly controlled by the Rb protein, which is often mutated or inactivated by hyperphosphorylation and thus quickly degraded in many cancers including sarcomas (reviewed in [91]). The ability of E2F to control the promoters of *MMP* genes couples Rb deregulation to the acquisition of metastatic properties by cancer cells. Interestingly, this mechanism seems to be utilized also by oncoviruses. The human papilloma virus (HPV) encodes for the E7 oncoprotein, which binds to and targets Rb for degradation thereby activating, among others, the E2F transcription factor. Since the ectopic expression of the E7 oncoprotein from the highly pathogenic HPV 16 strain in carcinoma cell lines upregulates the expression of MMP9, MMP14 and MMP13 [92], it is likely that this upregulation takes place through E2F. However, whether this is the molecular mechanism driving the increased invasiveness and metastasis of HPV-induced cervical and oropharyngeal carcinomas remains to be proven.

*MMP14* gene transcription can be regulated by different intra and extra-cellular pathological and physiological stimuli. Cells, when embedded in 3D collagen matrices, mimicking the sarcoma microenvironment (further discussed below), display increased levels of MMP14 [93,94]. The TF Egr1, which levels are increased by mechanical stimulation and can thereby be recruited to the *MMP14* promoter, has been identified as an MMP14 regulator in such collagenous microenvironments [93].

Another level of MMP14 regulation occurs through the DNA methylation of the MMP14 promoter which, like 70% of the human genes annotated so far, contains CpG islands [95,96]. DNA methylation patterns are crucial for the activity of methylation-sensitive TFs such as the MMP14 regulator Sp1. Both during differentiation and cancer, the affinity of this TF to its consensus DNA binding sequence can be modulated by DNA methylation [97,98].

It is becoming increasingly clear that, along with altered global gene expression, also the genomic distribution of methylated DNA sequences (called methylome) is distorted in malignant cells compared to that of their healthy counterparts [99]. In cancer cell lines, the methylation status of both *MMP14* and *MMP2* promoters is inversely correlated to their gene expression and to the cell migratory ability. Hypermethylated *MMP14* and *MMP2* promoters have been found in the non-invasive MCF7 breast cancer cell lines, whereas highly migratory glioma cells display hypomethylated promoters coupled to high MMP14 and MMP2 expression levels [100,101].

The cancer suppressor protein kinase D1 (PKD1), a repressor of different MMPs, including MMP14, is highly expressed in normal breast tissue, but epigenetically silenced by DNA methylation in invasive breast cancer, where MMP14 and other MMPs are induced [102]. Moreover, PKD1 is downregulated in invasive osteosarcoma compared to benign schwannoma, and PDK1 expression correlates with MMP levels and cell invasiveness [103].

### 5.2. Post-Transcriptional Regulation of MMP14

With respect to the regulation of mRNA, various microRNAs have been found to target the MMP encoding mRNAs, including those for MMP14, thus inhibiting their translation. Although several microRNAs have been reported to target MMP14, their activities in sarcoma remain uninvestigated. However, MMP14-targeting microRNAs, such as miR-193a-3p [104] and miR-133a [105] inhibit osteosarcoma proliferation, invasion, and metastasis [106,107]. The miRNA let-7 [108] is frequently downregulated in uterine leiomyosarcoma and Kaposi sarcoma [109,110], and the low expression of miR-34a [111] correlates with poor survival and response to chemotherapy in Ewing’s sarcoma patients [112].

**Figure 3:**
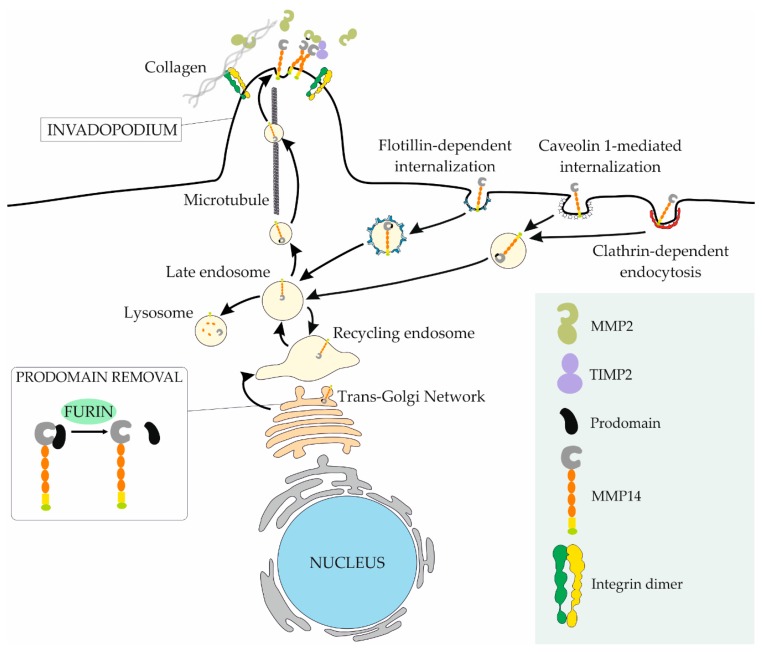
The plasma membrane expression of MMP14 is highly regulated. After translation, MMP14 is an inactive zymogen containing an inhibitory pro-domain that is cleaved in the Trans-Golgi Network by proprotein convertases (i.e., furin). Thereafter, MMP14 is transported to invadopodia, actin-rich protrusions with high matrix degradation capabilities, through microtubules. Internalization of MMP14 occurs via clathrin-, flotillin-, and/or caveolin 1-dependent endocytosis. The internalized MMP14 is then stored in late and recycling endosomes, where it can be re-directed to the plasma membrane, or degraded in lysosomes.

MMPs are translated as latent zymogens containing an N-terminal prodomain that acts as a shield for the catalytic site [113,114]. Removal of this inhibitory prodomain of MMP14 takes place largely in the trans-Golgi network by proprotein convertases including furin and PC6 in a membrane tethering-dependent manner [115,116,117]. However, alternative mechanism might also take place, for instance, autocatalytic processing activity of the MMP14 proenzyme has been reported in vitro [118]. In rhabdomyosarcoma, furin expression has been linked to malignancy in part due to its function in the processing of pro-MMP14, whereas in osteosarcoma, furin inhibition leads to reduced MMP14-dependent cell migration [119,120]. Thereafter, MMP14 intracellular trafficking to the plasma membrane requires an active exocytosis of MMP14-containing Rab8-positive vesicles dependent on microtubules and the motor proteins kinesins (Figure 3) [121,122].

At the cell surface, MMP14 is enriched in the invasive membrane protrusions known as invadopodia [123]. Localization of MMP14 to these invasive structures depends on various molecules that are essential for invadopodia formation such as cortactin, palladin, and the Rho-GTPases cdc42 and RhoA. Furthermore, MMP14 itself has been proposed to be necessary for the formation and maturation of these structures [124,125,126,127]. The control of MMP14 membrane expression is finely regulated by its internalization and recycling, with numerous molecules controlling the endosome-to-plasma membrane trafficking [128,129]. The internalization process of MMP14 is mediated by both clathrin-dependent and caveolae-dependent endocytosis leading to its transport to lysosomal degradation or recycling compartments from where it can relocate to the plasma membrane [130,131,132,133]. Additionally, MMP14 can be secreted at the membrane of small extracellular vesicles [134].

MMP14 internalization is regulated by the protein kinase c-mediated phosphorylation of its cytoplasmic tail [135]. Interestingly, a main component of caveolae, caveolin-1, has been reported to promote MMP14-dependent pro-MMP2 activation in Ewing sarcoma cells contributing to their metastatic potential, however, in epithelial breast cancer cells, but not in mesenchymal melanoma cells, caveolin-1 leads to a reduced cell migration due to the decreased secretion of MMP2 and MMP9 [136,137,138]. On the other hand, the membrane proteins flotillins, which form caeolin-1- and clathrin-independent membrane invaginations [139], promote MMP14 internalization inducing its recycling to invadopodia and ECM degradation in both high flotillin-expressing carcinoma and sarcoma cells [140]. This function may contribute to the association of flotillins with poor prognosis in various carcinomas and rhabdomyosarcoma [140].

### 5.3. MMP14 Regulation at the Membrane

The activity of MMPs at the plasma membrane is constantly regulated by the endogenous tissue inhibitors of metalloproteinases TIMPs, which reversibly bind to MMPs in a 1:1 stoichiometry [141]. There are four members of TIMPs (TIMP1-4) in humans, which show tissue-specific expression [142]. The balance between TIMP and MMP expression is important to maintain normal tissue homeostasis and is often dysregulated in cancer. TIMP2-4, but not TIMP1, are strong inhibitors of MMP14 [143]. TIMP2 forms a complex with pro-MMP2 that is recognized by one molecule of the MMP14 homodimer allowing the activation of pro-MMP2 by the second molecule of MMP14 [144,145,146,147]. Thus, although TIMP2 is important for pro-MMP2 activation, an excess of TIMP2 would inhibit both MMP14 molecules.

Gene expression of *TIMP2* in sarcomas is generally high. In Ewing sarcoma and rhabdomyosarcoma, however, *TIMP2* follows a similar pattern of low expression, as with *MMP14.* Interestingly, *MMP2* and *MMP14* genes are the first and third most significantly co-expressed genes with *TIMP2* in sarcoma (TCGA-SARC) indicating that these genes share common regulation mechanisms. Gene expression of *TIMP4* is low in sarcomas except for liposarcoma. Moreover, the general expression of *TIMP2* is upregulated in sarcomas compared to normal tissue (http://gepia.cancer-pku.cn). This upregulation has been specifically reported in alveolar soft-part sarcoma and chondrosarcoma [148,149]. At the protein level, TIMP2 expression correlates with that of MMP2 and MMP14 in chondrosarcoma, all three proteins displaying elevated levels in high-grade anaplastic components compared to low-grade components of de-differentiated and conventional chondrosarcoma [150]. In synovial sarcoma, however, low expression of TIMP2 is a poor prognostic factor for disease-free survival [70]. TIMP4 expression in turn is relatively high in well-differentiated liposarcoma and low in the more aggressive undifferentiated liposarcoma, whereas TIMP1 shows an opposite expression pattern in these sarcomas. The switch from well-differentiated to undifferentiated phenotypes occurring at low TIMP4 and high TIMP1 levels has been attributed to the activation of the oncogenes yes-associated protein (YAP) and transcriptional co-activator with PDZ binding motif (TAZ), which have also been associated with sarcomagenesis and maintenance of stem cell-like features in various cancer types [151,152,153].

MMP14 activity can also be inhibited by other proteins. Reversion-inducing-cysteine-rich protein with kazal motifs (RECK) is a membrane-anchored inhibitor of MMP14 [154]. In addition, testican 3 and N-Tes (a splicing variant of testican 3) have inhibitory effects on MMP14 and MMP2 activation [155]. On the other hand, the tight junction proteins claudins have been shown to induce MMP14-dependent activation of pro-MMP2 independently of TIMP2, a process not restricted to the cell–cell border [156,157]. Tight junctions are typically formed in epithelial and endothelial cells, but some sarcoma cells including synovial sarcoma and osteosarcoma as well as osteoblasts form these structures [158,159,160]. De-localization of claudin-1 from tight junctions has been observed in metastatic osteosarcoma cells [160], suggesting its involvement in metastasis could be linked to MMP14 activity.

### 5.4. ECM Biomechanics and Dimensionality Affect MMP14

ECM stiffness has been linked to aggressiveness and EMT in various cancer cell types, and sensors of the mechanical properties of the ECM such as integrins are important players in MMP14-mediated cell invasion in 3D microenvironments [161]. Several studies have investigated the effects of ECM stiffness on MMP14 expression and activity. However, most of the models used to date consist of stiffness-controlled 2D substrates. These studies, which include carcinoma cells and mesenchymal cells, fail to show a clear link between stiffness and *MMP14* gene expression and activity [94,162,163,164,165,166]. The observed variability may be explained by the uncoupling of ECM stiffness and MMP14 activity in cancer. This deregulation has been reported when comparing endometrial stromal cells and endometriotic stromal cells, where the former show enhanced *MMP14* gene expression with substrate stiffness but the latter are unresponsive [166]. Moreover, in carcinoma cells, substrate stiffness has been linked to EMT, which could lead to indirect changes in MMP14 expression [167].

The activity of MMP14 is highly relevant in 3D environments where physical constrictions impede the free migration of cells. Moreover, the expression and activity of MMP14 is enhanced when cells are in 3D matrices compared with 2D, even when the stiffness of these are comparable, an increase that was reported to be independent of β1-integrin binding with the ECM [94]. Most models that have been used to study the function of MMP14 in 3D microenvironments have used collagen matrices of various concentrations and crosslinking conditions. However, these models do not systematically control pore size, integrin ligand density, and collagen fiber mechanical properties, all factors that can potentially affect MMP14. Thus, the use of engineered matrices controlling integrin ligand and cleavable site density, as well as porosity and mechanical properties will shed light on the regulation of MMP14 by the different factors independently.

Recent studies have shown that both force- and MMP-dependent matrix remodeling regulate confined cell migration and cell phenotype, indicating that both mechanical and biochemical properties of the cellular milieu affect cell behavior [168,169]. These effects could be involved in the relationship between MMP14 expression and the mesenchymal phenotype, where changes in cell confinement and the ability to remodel the ECM as a result of MMP14 activity could lead to phenotypical changes in sarcoma cells.

## 6. Sarcoma Metastasis and MMP14

Cancer cell dissemination and colonization of distant organs coupled to therapy resistance is the main cause of mortality associated to cancer. Dissemination of sarcoma cells from the primary tumor to secondary sites occurs through three main distinct routes, the vascular system, the lymphatic system, or directly into tissues and body cavities. The unique relationship between sarcoma cells and their milieu is fundamental to understand the characteristic metastatic process of these cells, with MMPs playing a central role [9].

### 6.1. ECM Architecture and MMP14 in Cell Migration

To initiate the metastatic process, sarcoma cells dissociate from the primary tumor, gaining the ability to invade benign/stromal tissues. Cancer cells can use various modes of migration to invade their surroundings depending on their cell/tissue of origin and phenotype as well as the characteristics of their microenvironment. Cells migrate through the ECM by remodeling it, opening migration tracks or by using pre-existing tracks [170]. When pore-like migration tracks are too small for a cell to move through, proteolytic degradation of the ECM is often required [171]. In vitro studies show that fibrosarcoma HT-1080 cells embedded in high-density collagen matrices, which have small pores, induce collective migration, a migration type that consists of cells invading together following initial leader cells [172]. This process is dependent on the MMP-mediated proteolytic collagen breakdown. Although MMP14 is required for HT-1080 cell migration in dense collagen matrices, the directionality of their migration is driven by the fibrillar topography of the ECM independently of MMP14, which further indicates the strong effect of the ECM architecture on cell migration [173].

**Figure 4:**
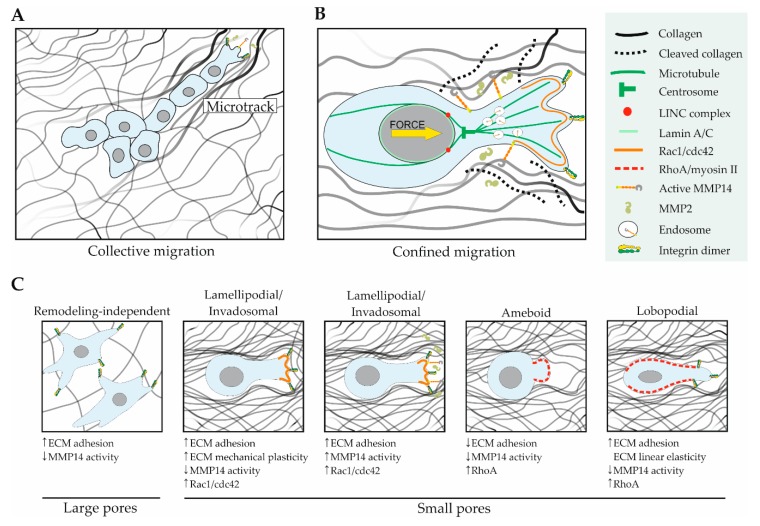
The structure and mechanical properties of the ECM, as well as MMP14 activity can all affect the mode of 3D cell migration. (**A**): Mesenchymal cells embedded in a dense 3D collagen matrix migrate collectively due to the formation of microtracks initiated by the MMP14-dependent collagenolysis first induced by leader cells. (**B**): In mesenchymal cells migrating in matrices with small pores (confined environment), MMP14-rich endosomes are polarized in front of the nucleus to direct the matrix degradation pericellularly towards the direction of the migration enabling the formation of pores wider than the cell nucleus. This endosome polarization depends on the LINC complex and the positioning of the centrosome ahead of the nucleus. (**C**): The type of 3D cell migration depends on characteristics of the matrix such as pore size and its elasticity and plasticity but also depend on cellular features such as the activity of MMP14, and that of the Rho GTPases Rac1, cdc42 impacting on the formation of invadopodia and matrix degradation, and RhoA inducing myosin II-dependent contractility.

Recently, several models have been proposed to explain the mechanisms governing mesenchymal cell migration in dense collagen matrices. Regarding collective cell migration, a study using fibrosarcoma HT-1080 cells shows that these cells present an anterior force-generating leading edge containing β1-integrin, F-actin, and MMP14 responsible for the re-alignment of collagen fibers into microtracks, which cells use to migrate. The following cells also use these tracks and further widen them, as they are the sites of least confinement, resulting in collective migration [161] (Figure 4A). A second study links cell migration in confined microenvironments with localized MMP14-dependent collagenolysis and the cell nucleus (Figure 4B). A limiting factor in confined migration, where the diameter of pores is smaller than that of the nucleus, is the capacity of the nucleus to deform [174]. In migrating mesenchymal cells, MMP14-containing storage endosomes become polarized in front of the nucleus in the direction of cell migration and their positioning depends on the function of the linker of nucleoskeleton and cytoskeleton (LINC) complex in connecting the nucleus and the centrosome [175]. Therefore, the nucleus can act as a sensor of the ECM architecture to direct collagenolysis. Nuclear deformation depends on its stiffness and, interestingly, loss of MMP14 causes alterations of the nuclear envelope and nuclear softening, further indicating a relationship between MMP14 and the nucleus [176].

The mode of migration used by mesenchymal cells is determined by both the structural and mechanical characteristics of the matrix, and by the ability of cells to remodel it (Figure 4C). Matrix degradation is not required for mesenchymal migration when the pore size of the matrix is large and when the matrix is mechanically plastic, which allows cells to deform it by applying force [169]. This type of migration is characterized by the formation of large protrusions at the leading cell edge called lamellipodia or by invadopodia. On the other hand, inhibition of MMP activity in HT-1080 cells embedded in dense 3D matrices causes decreased adhesion and increased actomyosin activity, leading to cell rounding and a switch of the migration type to ameboid [177]. Healthy primary fibroblasts switch their migration type from lamellipodial to a high pressure lobopodial-forming type in highly crosslinked collagen matrices. Lobopodial migration occurs in matrices presenting linear elasticity and in cells with high RhoA-ROCK-myosin II activity [178]. Similarly, fibrosarcoma HT-1080 and SW684 cells switch to a high-pressure lobopodial migration after MMP pharmacological inhibition. This migration mode depends on integrin adhesion, actomyosin contractility, and the LINC complex protein Nesprin-3 [179]. Altogether, these studies demonstrate the dependence of mesenchymal cells on MMP14 activity and the physical characteristics of the extracellular milieu to determine the mode of migration.

### 6.2. MMP14 and the Vascular Metastatic Route

Contrary to carcinomas, which actively metastasize to lymph nodes via lymphatic vessels (lymphogenous route), sarcomas primarily spread through blood vessels (hematogenous route), except for epithelioid sarcoma, clear cell sarcoma, angiosarcoma, and alveolar rhabdomyosarcoma [180,181]. Subsequently, the hematogenously spreading soft tissue sarcomas and osteosarcoma predominantly metastasize to the lungs [182,183].

Knock-down of MMP14 in triple negative breast cancer cells, which have a mesenchymal phenotype, reduces blood vessel invasion but not lymphatic vessel invasion. Moreover, MMP14 expression in cancer cells induces the expression of MMP14 in blood but not in lymphatic endothelial cells [184]. Downregulation of MMP14 also reduces the formation of lung metastases without affecting primary tumor size in a mouse model of breast cancer [185]. Furthermore, collectively migrating cells, but not singly-migrating cells, are restricted to lymphatic invasion [185]. In line with these observations, we reported that reduction of MMP14 membrane expression caused by MMP16, the other MT-MMP frequently expressed in sarcomas (http://ist.medisapiens.com/) forming complexes with MMP14 [186], promotes collective migration in melanoma cells that preferentially invade lymphatic vessels [187]. This suggests that MMP14 and a mesenchymal-like single cell migration facilitates the blood vessel invasion of these cells. Although mechanistic studies on the preference for sarcoma cells to use the hematogenous over the lymphogenous route, and to metastasize in the lungs, are lacking, the mesenchymal characteristics and the high MMP14 expression of these tumors might explain the intravasation into blood vessels. Moreover, the low MMP14 expression of alveolar rhabdomyosarcomas and the epithelial characteristics of epithelioid sarcomas is consistent with their preference for utilizing the lymphogenous route.

## 7. Clinical Implications and Future Perspectives

Matrix metalloproteases, including MMP-14, are attractive therapeutic target candidates due to central functions in numerous diseases, as well as their principally druggable cell surface or extracellular activities. However, more than 50 MMP inhibitors have been investigated in various clinical trials without success [188]. Several small molecule inhibitors of MMP14 have been developed, but so far none of them has succeeded in clinical trials. The failure of these clinical trials has been attributed to several factors, such as the biological complexity of the MMP function or the inhibitors themselves, as well as poor trial design with inadequate clinical endpoints. There are, however, ongoing attempts to develop new, more precisely targeted, MMP inhibitors. For example, novel inhibitory anti MMP14 antibodies have been recently developed and used in cell lines and primary xenograft assays to limit hypoxia, immune suppression, and metastasis [189]. Another recent study has demonstrated efficacy of targeting the MMP14/MMP2/integrin αvβ3 axis using protein monomers and heterodimers that bind to both MMP14 and integrin αvβ3 [190]. These studies demonstrate that interest in therapeutic targeting of MMP14 continues.

Understanding the specific alterations and functions of MMP14 and its regulation in sarcomas can open new opportunities to find targetable candidates to inhibit sarcoma invasion and metastasis. However, as seen in pediatric sarcomas, MMP14 expression is not necessary for sarcoma development and progression, and its inhibition in high MMP14-expressing sarcomas may not be effective to block sarcoma cell dissemination or disease progression. This could be due to the activation of alternative mechanisms of cancer invasion and metastasis [177,179]. Investigating the differences between low- and highly-expressing MMP14 sarcoma types in invasion and metastasis will shed light on these questions. Moreover, as the ECM characteristics influence cell behavior and MMP14 activity, targeting ECM changes occurring during tumor development, a process known as ECM normalization, could be beneficial for these patients. Currently, several promising ECM-normalizing and mechanotransduction-targeting drugs are in clinical trials, thus, it will be of interest to investigate whether these affect MMP14 function as part of their mechanisms of action [191]. Furthermore, the changes in the ECM occurring during development and aging could be involved in the strong differences observed between pediatric and adult sarcomas [192].

In terms of use as a biomarker, the biomarkers that achieve most clear breakthrough into diagnostics are typically those related to a specific therapy. This bar is high and has yet to be met by MMP14. Further studies are required to conclude whether MMP14 overexpression alone, or possibly in combination with markers of ECM alterations or other MMP14 target proteins, may indicate particular tumor subtypes that could then be treated with a more molecular-based therapy. Considering the poor correlation between MMP14 gene and protein expression and its activity, the potential of using the active form of MMP14 or its effectors as biomarkers should also be further explored. Additionally, as MMP14 function is highly regulated by the microenvironment, active MMP14 quantification should preferentially be performed within the native tissue context. To meet this need, several fluorescent and bioluminescent probes have been developed [193]. The use of these probes and novel MMP activity reporter constructs will facilitate the investigation of the relationship between MMP14 and the tumor microenvironment in vivo [194].
